# Harnessing Machine Learning for Prediction of Postoperative Pulmonary Complications: Retrospective Cohort Design

**DOI:** 10.3390/jcm12175681

**Published:** 2023-08-31

**Authors:** Jong-Ho Kim, Bo-Reum Cheon, Min-Guan Kim, Sung-Mi Hwang, So-Young Lim, Jae-Jun Lee, Young-Suk Kwon

**Affiliations:** 1 Department of Anesthesiology and Pain Medicine, Chuncheon Sacred Heart Hospital, Hallym University College of Medicine, Chuncheon 24253, Republic of Korea; poik99@hallym.or.kr (J.-H.K.); qhxoddl15@hallym.or.kr (B.-R.C.); soojeongsun@hallym.or.kr (M.-G.K.); h70sm@hallym.or.kr (S.-M.H.); inooim@hallym.or.kr (S.-Y.L.); iloveu59@hallym.or.kr (J.-J.L.); 2 Institute of New Frontier Research Team, Hallym University College of Medicine, Chuncheon 24252, Republic of Korea

**Keywords:** surgery, lung, complications, machine learning, prediction

## Abstract

Postoperative pulmonary complications (PPCs) are significant causes of postoperative morbidity and mortality. This study presents the utilization of machine learning for predicting PPCs and aims to identify the important features of the prediction models. This study used a retrospective cohort design and collected data from two hospitals. The dataset included perioperative variables such as patient characteristics, preexisting diseases, and intraoperative factors. Various algorithms, including logistic regression, random forest, light-gradient boosting machines, extreme-gradient boosting machines, and multilayer perceptrons, have been employed for model development and evaluation. This study enrolled 111,212 adult patients, with an overall incidence rate of 8.6% for developing PPCs. The area under the receiver-operating characteristic curve (AUROC) of the models was 0.699–0.767, and the f1 score was 0.446–0.526. In the prediction models, except for multilayer perceptron, the 10 most important features were obtained. In feature-reduced models, including 10 important features, the AUROC was 0.627–0.749, and the f1 score was 0.365–0.485. The number of packed red cells, urine, and rocuronium doses were similar in the three models. In conclusion, machine learning provides valuable insights into PPC prediction, significant features for prediction, and the feasibility of models that reduce the number of features.

## 1. Introduction

Pulmonary complications are the main causes of postoperative morbidity and mortality [1]. The reported incidence of postoperative lung complications varies depending on the patient population and the criteria used to define the complications [2]. Postoperative pulmonary complications (PPCs) include almost all complications affecting anesthesia and the postoperative respiratory tract. These complications are heterogeneous and commonly defined, have significant adverse effects on patients, and are difficult to predict [3]. Predicting PPCs can play an important role in postoperative patient care because it reduces the risk of potentially serious complications and allows doctors to plan appropriate strategies. By identifying patients with potential PPC outbreaks, health-care providers can prepare alternative technologies, equipment, and personnel to ensure successful postoperative patient care and avoid complications, such as ventilator care, intensive care, and death. Thus, for PPC management, preventive strategies may be more effective than treating established PPCs. Identifying the risk of PPCs before surgery is important to guide preventive interventions to reduce the risk and incidence of PPCs [4,5].

Machine learning (ML), a subfield of artificial intelligence (AI), has shown remarkable potential in various medical applications, including predictive analytics [6,7,8,9]. Using ML algorithms, health-care providers can analyze large volumes of patient data, identify patterns, and generate accurate predictions [10]. This study focuses on the possibility of utilizing ML techniques to predict postoperative pulmonary complications to aid clinicians in risk assessment, conduct early intervention, and improve patient outcomes.

## 2. Materials and Methods

### 2.1. Data Collection

This retrospective cohort study protocol was approved by the Clinical Research Ethics Committee of Chuncheon Sacred Heart Hospital, Hallym University. The need for informed consent was waived due to the retrospective study design. The medical records of patients treated between 1 January 2011 and 15 November 2021 were obtained from the clinical data warehouses of two hospitals affiliated with Hallym University Medical Center. One hospital (Hallym University Sacred Heart Hospital) is located in a metropolitan area, and the other (Chuncheon Sacred Heart Hospital) is located in a nonmetropolitan area.

### 2.2. Patients and Postoperative Pulmonary Complications

This study included adult patients aged ≥ 18 years who did not exhibit any preoperative pulmonary complications. Patients with missing or outlier data were excluded. PPCs include atelectasis, pulmonary edema, pleural effusion, pneumothorax, pulmonary embolism, respiratory failure, pneumonia, and acute respiratory distress. The determination of pulmonary complications is presented in Supplementary Table S1. All radiological findings were confirmed by radiologists.

### 2.3. Dataset

The dataset included 102 perioperative variables, including patient characteristics, preexisting diseases, and intraoperative factors. The definitions of the variables are summarized in Supplementary Table S2. Before model learning, the datasets were standardized using min–max scaling. The dataset was divided into training and test datasets. The training set comprised 20% of the total dataset. The training and test sets were stratified so that they included PPCs at the same rate.

### 2.4. Machine Learning

During supervised learning, an ML method was used to infer a function from the training data, and a classification method was used to mark the value of a given input vector. We used five ML algorithms, which contained the following: logistic regression, random forest, light-gradient boosting machine, extreme-gradient boosting machine, and multilayer perceptron (MLP) [11,12,13,14,15,16,17,18].

Developing classification models using imbalanced datasets poses the risk of yielding ineffectual models that exhibit an inability to accurately classify minority classes of substantive interest. Moreover, a small number of observations in a minority class is insufficient to represent a small number of observations that sample the population distribution of a minority group, thus risking overfitting the classification model. Therefore, even if minority groups are classified in the training dataset, there is a risk that they will not be classified properly in the new data. Therefore, we use the synthetic minority oversampling technique for all algorithms [19].

**Figure 1:**
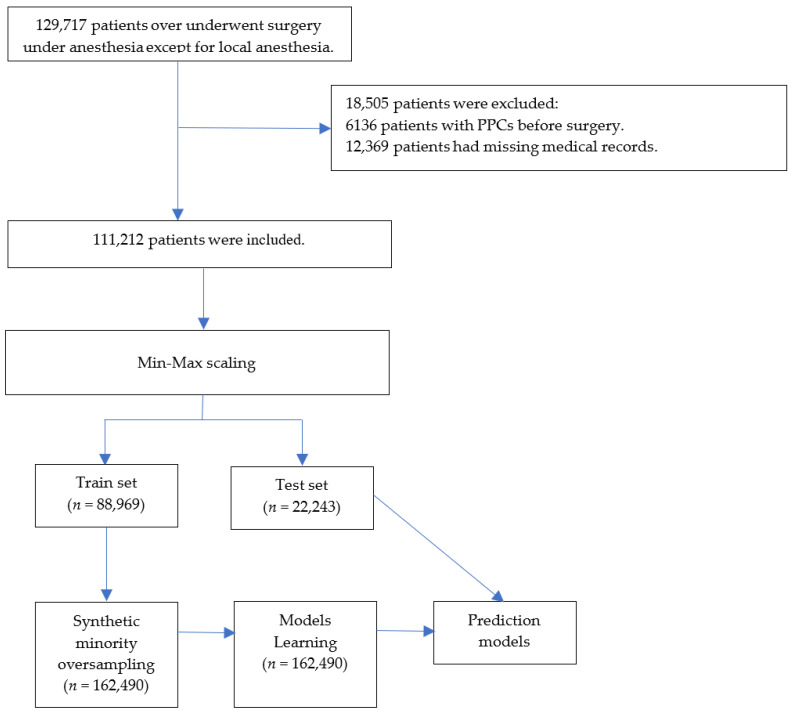
Flowchart. PPCs = postoperative pulmonary complications.

ML models have hyperparameters that must be set to customize the model to the dataset. Although the general effects of hyperparameters on models are known, establishing a combination of hyperparameters that interact with them in a given dataset is difficult. A better approach is to objectively search for different values of model hyperparameters and select a subset that generates models that achieve the best performance on a given dataset. This is known as hyperparameter optimization or hyperparameter tuning [20]. We used a random search for hyperparameter optimization [16], in which each dimension represents a hyperparameter and each point represents a single model configuration, defining the volume to be searched as a restricted domain of hyperparameter values and random samples from that domain. The data processing and the ML processes are summarized in Figure 1.

### 2.5. Feature Importance and Simplified Models

The feature importance of the models was obtained using a built-in function after a random search for hyperparameter optimization. Models with ten important features were created and evaluated to assess the performance of the simplified models for practicality.

### 2.6. Statistics and Metrics

Descriptive analyses were performed to compare the characteristics and perioperative data of patients with and without PPCs. Categorical variables are presented as numbers (%), and continuous variables are presented as medians (interquartile ranges). Differences were evaluated as absolute standardized differences. Five metrics were calculated to assess model performance. The area under the receiver-operating characteristic curve (AUROC) was used as a metric. The recall, precision, f1 score, and accuracy were calculated. Bootstrapping (*n* = 20,000) was performed to calculate 95% confidence intervals. Python (version 3.7; Python Software Foundation, Beaverton, OR, USA) was used to calculate model metrics.

## 3. Results

### 3.1. Patient Characteristics

This study enrolled 129,717 patients aged ≥ 18 years who did not exhibit preoperative pulmonary complications. After excluding 18,505 patients with missing (*n* = 12,369) and preoperative pulmonary complication (*n* = 6136) data, 111,212 patients were included in model development and evaluation (Figure 1). The patient characteristics and perioperative data are summarized in Table 1 and Table 2. Among the encompassing array of patient characteristics and perioperative data, those features demonstrating an absolute standardized difference of less than 0.1 were as follows: Male, body mass index, hypothyroidism, peptic ulcer disease, acquired immunodeficiency syndrome/human immunodeficiency virus, lymphoma, rheumatoid arthritis/collagen vascular diseases, obesity, weight loss, blood loss, anemia, drug abuse, psychoses, alcohol, smoking amount, inhalation anesthetics, N_2_O, intraoperative cryoprecipitate, succinylcholine, pethidine, activated partial thromboplastin time, uric acid, laparoscopic surgery, and musculoskeletal surgery, urogenital surgery.

The percentage of patients who developed PPCs was 8.6%. The training set included 88,969 patients and was oversampled from 162,490 patients. The test set comprised 22,243 patients.

### 3.2. Model Performance

The AUROC of the models was 0.699–0.767, and that of the logistic regression model was the highest. The F1 score of the models was 0.446–0.456, and that of the random forest model was the highest. The details of the model performance are summarized in Table 3.

### 3.3. Feature Importance

**Figure 2:**
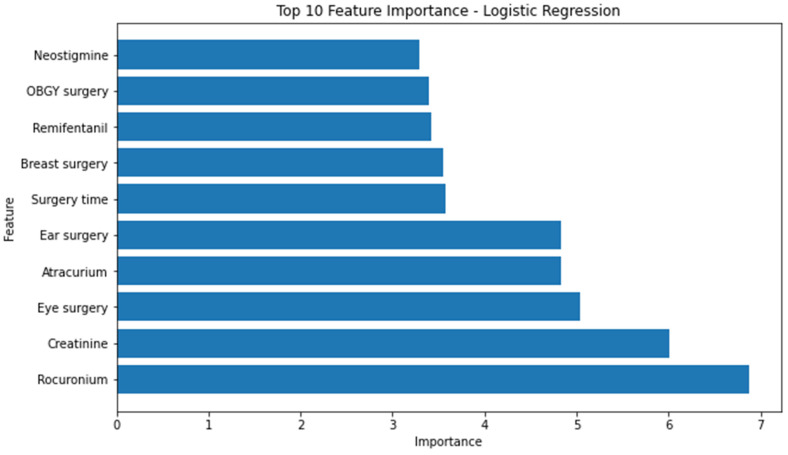
Top 10 feature importance of logistics regression model. OBGY = obstetrics and gynecology.

Using the built-in function, we obtained the feature importance of each model except the MLP model. The ten most significant attributes extracted from the logistic regression model, characterized by the highest area under the receiver-operating characteristic curve (AUROC), encompassed the following variables: rocuronium, creatinine, eye surgery, atracurium, ear surgery, duration of surgery, breast surgery, administration of remifentanil, obstetrics and gynecology surgery, and administration of neostigmine (Figure 2).

**Figure 3:**
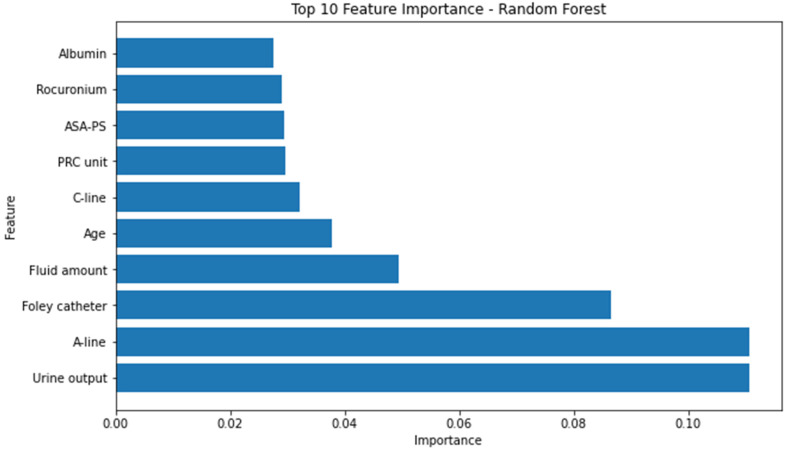
Top 10 feature importance of random forest model. A-line = arterial continuous monitoring line, ASA-PS = American Society of Anesthesiologists physical status, C-line = central venous pressure monitoring line, PRC = packed red cell.

Conversely, the attributes identified as the most influential within the random forest model, as determined by the highest F1 score, consisted of the following factors: urine output, utilization of arterial continuous monitoring line, presence of foley catheter, volume of administered fluids, patient age, employment of central venous pressure monitoring line, transfusion of packed red blood cells, American Society of Anesthesiologists physical status classification, administration of rocuronium, and serum albumin levels. (Figure 3) The significance of the arterial continuous monitoring line stood out conspicuously in comparison to other attributes within the XG boosting model. Notably, albumin, sodium, and potassium, among others, emerged as pivotal features in the light-gradient boosting machine. A comprehensive overview of the feature importance rankings for both the XG boosting and light-gradient boosting machine models is provided in Appendix A and Appendix B, respectively.

**Figure 4:**
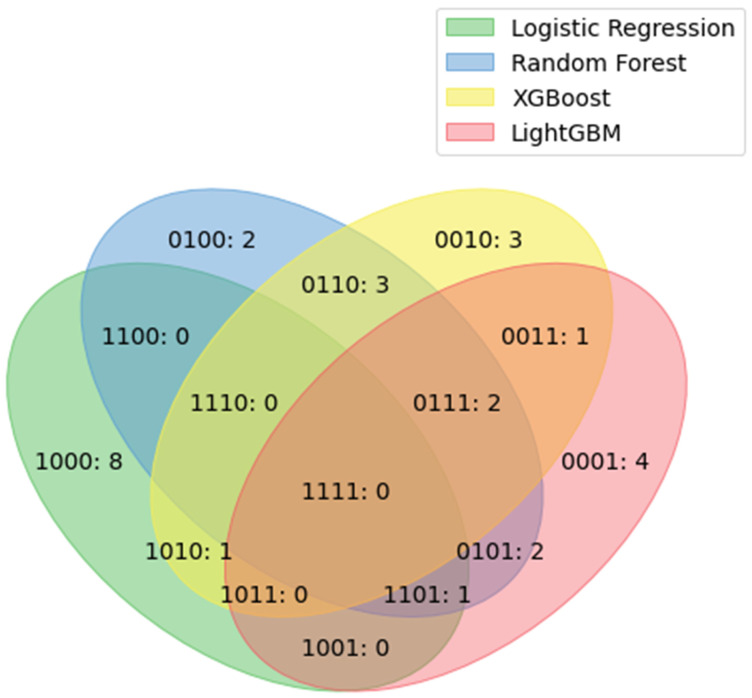
Duplicated features among the top 10 important features of each model. The four numbers represent the logistic regression, random forest, XG boosting, and LGBM models, respectively, in order from the beginning. Zero indicates none, and 1 indicates present. 1101: rocuronium; 0111: urine output and PRC unit. LGBM = light-gradient boosting machine, XG = extreme gradient, PRC = packed red cell.

Figure 4 lists the important duplicated features of the models. No features among the top 10 features were included in all of the models. Three features were included in the top 10 important features of the three models: rocuronium, urine output, and packed red cells (PRCs).

### 3.4. Evaluation of Simplicity Model

Table 4 shows the evaluation of models, including the top 10 features. Generally, the performance of the simplicity model is lower than models including all features. However, AUROC is highest in the random forest model, unlike the results of models including all features.

## 4. Discussion

This cohort study used five ML algorithms with 102 features, including preoperative and intraoperative data, to predict the occurrence of PPCs. Furthermore, we identified the important factors according to the algorithm and developed simpler models that included the top 10 important features. Some factors are common as the major features of several models. Although the accuracy of the prediction models was high, metrics such as AUROC and f1 score were not excellent in all models.

The area of prediction of PPCs using AI has been extended. Recent studies have demonstrated the emergence of a new paradigm. However, previous studies were limited to specific groups or pulmonary diseases. Peng et al. conducted a multicenter study that focused on geriatric patients and utilized a deep neural network model to predict PPCs [7]. Although it provides valuable insights into a specific patient population, its generalizability to other age groups and surgical specialties remains uncertain. Xue et al. developed a model using preoperative and intraoperative data to identify the risk of postoperative pneumonia [8]. Their study investigated the use of ML to predict various postoperative complications, including pneumonia, among PPCs. However, they did not specifically highlight the distinct characteristics and risk factors associated with PPCs other than pneumonia, which potentially diluted the focus on PPCs. Xue et al. focused on predicting pulmonary complications, specifically in emergency gastrointestinal surgery [9]. However, this study did not explore the broader landscape of PPCs across different surgical procedures and settings.

In this study, we aimed to address the limitations of previous research and provide novel insights into the prediction of PPCs using ML techniques. Our study’s primary outcome focused exclusively on PPCs, allowing for a comprehensive evaluation of the risk factors specific to this complication. We collected a large and diverse dataset of patients with various surgical specialties, age, and comorbidity profiles. This enabled a more comprehensive understanding of the risk factors associated with PPCs in different patient populations. We employed advanced feature-selection techniques to identify the most relevant predictors of PPCs, ensuring optimal model performance and reducing the potential for overfitting. This approach can enhance the accuracy and generalizability of predictive models [21,22]. However, despite the development of a simplified model, it did not exhibit better performance. Although we used a method to enhance performance, such as oversampling, hyperparameter tuning, data preprocessing, and min–max scaling, it was insufficient to obtain the best performance.

The choice of features or variables used in models can significantly affect their performance. If the selected features do not capture the relevant patterns or contain noise, suboptimal performance can be achieved [23]. Using only the top 10 features may not capture the relevant patterns of PPCs. In our models, the top 10 features were usually considered factors associated with PPCs. In this study, urine output [24,25], transfusion of red blood cells [26,27], and rocuronium [28] were the top 10 important features in three models, and most models have several features associated with PPC as the top 10 important features. This suggests that ML algorithms may use generally known factors associated with PPC. To improve the performance in future studies, we can add data to these features or consider enhanced feature engineering techniques.

Machine learning, a constituent of artificial intelligence, has showcased immense potential across diverse medical domains, notably in predictive analytics. This technological facet holds the capacity to revolutionize health care by amplifying the precision of diagnoses, treatment strategies, and overall patient well-being. An area of profound application lies in intraoperative management, with specific emphasis on anesthesia depth and hemodynamics, resulting in notable reductions in postoperative complications. In the context of anesthesia depth regulation, AI emerges as a pivotal player, ensuring patient safety and optimizing anesthesia administration during surgical procedures. By adeptly analyzing an array of physiological parameters, AI algorithms dynamically calibrate anesthesia dosages, mitigating the risks of both insufficient and excessive sedation. Consequently, this proactive management approach contributes to the minimization of complications linked to anesthesia administration. Leveraging techniques such as machine learning and signal processing, AI effectively discerns patients’ anesthesia responsiveness, affording anesthesiologists the means to finely adjust drug delivery, thereby maintaining optimal levels throughout surgery [29,30]. Hemodynamics, another critical aspect, also shows substantial AI potential for refining intraoperative blood pressure and circulatory management, translating to diminished postoperative complications. Through real-time amalgamation of diverse data streams, AI algorithms provide valuable insights into patients’ cardiovascular status, enabling timely interventions to uphold stable hemodynamics and consequently abate the prospects of adverse postoperative outcomes. By scrutinizing patient-specific variables and perpetually monitoring vital indicators, AI prognosticates the likelihood of hypotensive episodes, empowering medical teams to adopt preemptive measures and alleviate potential complications [31,32,33]. In a holistic evaluation of patient data and the employment of predictive models, AI may serve as a tool for clinicians to navigate judicious decisions concerning fluid administration and cardiovascular support, ultimately culminating in heightened patient outcomes. The convergence of AI and medical science is expected as a transformative juncture, holding substantial promise for advancing the standards of intraoperative care and fortifying postoperative convalescence.

Although our study on the prediction of postoperative pulmonary complications using ML provided valuable insights, acknowledging its limitations is crucial. These limitations include the following. First, the quality and availability of the dataset used in our study may impact the generalizability of the results. If the dataset is limited in terms of sample size, representation of diverse patient populations, or specific surgical procedures, predictive models may not accurately capture the complexities and variability of PPCs across different settings. Furthermore, the dataset used in our study may not reflect the current medical practices and advancements, potentially affecting the applicability of our findings to contemporary health-care settings [34]. Second, while our study conducted internal validation procedures, including cross-validation, external validation of independent datasets is essential to assess the generalizability of the predictive models. If external validation is not performed, or the results from the external validation dataset are not reported, the reliability and robustness of our models may be uncertain [35]. Third, while our study included a substantial number of patients, it is important to acknowledge that the data were drawn from a single center and the study was retrospectively conducted. This may raise questions about the diversity of patient populations and medical practices, potentially affecting the generalizability of the models to other health-care settings. Additionally, the retrospective nature of the study introduces potential sources of bias and limitations in data collection. Finally, ML models predict outcomes; however, they may not provide causal explanations or elucidate the underlying mechanisms of the predicted outcomes [36]. Although our study identified the risk factors associated with PPCs, it may not definitively establish causality or provide deep insights into the biological or physiological pathways involved when considering the present performance of our models.

Our study represents an advancement in the prediction of PPCs using ML. By specifically addressing the limitations of previous research and providing novel insights into this complication, we contribute to the development of more accurate and clinically relevant prediction models, optimize resource allocation, and have the potential to guide perioperative management strategies for mitigating the risk of PPCs. However, obstacles to the realization of prediction models remain, and further studies are needed to overcome them.

## Figures and Tables

**Table 1:** Patient characteristics.

	Non-PPCs (*n* = 101,557)	PPCs (*n* = 9655)	ASD
Age	52 (39, 63)	67 (56, 77)	0.92
Male sex	49,313 (48.6)	5144 (53.3)	0.09
Order of surgery	1 (1, 1)	1 (1, 1)	0.19
Cooperative surgery (count)	1 (1, 1)	1 (1, 1)	0.13
Body mass index	24.2 (21.9, 26.7)	24 (21.5, 26.6)	0.07
Congestive heart failure	2910 (2.9)	1021 (10.6)	0.33
Cardiac arrhythmia	3846 (3.8)	890 (9.2)	0.23
Valvular diseases	601 (0.6)	247 (2.6)	0.17
Pulmonary circulation disorders	440 (0.4)	298 (3.1)	0.22
Peripheral vascular disorders	2200 (2.2)	442 (4.6)	0.14
Hypertension, uncomplicated	11471 (11.3)	2189 (22.7)	0.31
Hypertension, complicated	5033 (5.0)	971 (10.1)	0.20
Paralysis	424 (0.4)	137 (1.4)	0.11
Other neurological disorders	3310 (3.3)	759 (7.9)	0.21
Chronic pulmonary diseases	9176 (9.0)	1526 (15.8)	0.21
Diabetes, uncomplicated	6374 (6.3)	1218 (12.6)	0.22
Diabetes, complicated	6471 (6.4)	1352 (14.0)	0.26
Hypothyroidism	2225 (2.2)	327 (3.4)	0.07
Renal failure	3597 (3.5)	1022 (10.6)	0.29
Liver disease	4710 (4.6)	847 (8.8)	0.17
Peptic ulcer disease (excluding bleeding)	1853 (1.8)	272 (2.8)	0.07
AIDS/HIV	22 (0.0)	1 (0.0)	0.01
Lymphoma	402 (0.4)	76 (0.8)	0.05
Metastatic cancer	1112 (1.1)	276 (2.9)	0.13
Solid tumor without metastasis	17,016 (16.8)	3429 (35.5)	0.44
Rheumatoid arthritis/collagen vascular diseases	2532 (2.5)	289 (3.0)	0.03
Coagulopathy	697 (0.7)	209 (2.2)	0.13
Obesity	845 (0.8)	78 (0.8)	0.00
Weight loss	338 (0.3)	63 (0.7)	0.05
Fluid and electrolyte disorders	2621 (2.6)	844 (8.7)	0.28
Blood-loss anemia	312 (0.3)	25 (0.3)	0.01
Deficiency anemia	3012 (3.0)	611 (6.3)	0.16
Alcohol abuse	2106 (2.1)	370 (3.8)	0.11
Drug abuse	1647 (1.6)	193 (2.0)	0.03
Psychoses	660 (0.6)	128 (1.3)	0.07
Depression	5449 (5.4)	869 (9.0)	0.14
Unconsciousness	1780 (1.8)	1490 (15.4)	0.56
Alcohol (consumption/unknown)	27,481 (27.1)/4774 (4.7)	2038 (21.1)/584 (6.0)	0.03
Smoking amount (packs)	0 (0, 0)	0 (0, 0)	0.03
Smoking duration (years)	0 (0, 0)	0 (0, 0)	0.15
Emergency	15,986 (15.7)	2828 (29.3)	0.33
ASA-PS			1.08
1	30,415 (29.9)	419 (4.3)	
2	51,757 (51.0)	3254 (33.7)	
3	18,367 (18.1)	5022 (52.0)	
4	969 (1.0)	908 (9.4)	
5	37 (0.0)	52 (0.5)	
6	12 (0.0)	0 (0.0)	

AIDS = acquired immunodeficiency syndrome, ASA-PS = American Society of Anesthesiologists physical status, ASD = absolute standardized difference, HIV = human immunodeficiency virus, PPCs = postoperative pulmonary complications.

**Table 2:** Perioperative data.

	Non-PPCs (*n* = 101,557)	PPCs (*n* = 9655)	ASD
General anesthesia	87,884 (86.5)	9160 (94.9)	0.30
Inhalation anesthetics	85,174 (83.9)	8062 (83.5)	0.01
N_2_O	13,982 (13.8)	1274 (13.2)	0.02
Anesthesia time (min)	100 (65, 150)	175 (115, 270)	0.82
Surgery time (min)	65 (35, 110)	125 (75, 210)	0.77
Intraoperative fluid administration	450 (300, 800)	1400 (700, 2400)	0.93
Intraoperative urine output	0 (0, 15)	150 (35, 400)	0.63
Arterial line	26,167 (25.8)	8466 (87.7)	1.62
Central venous line	5978 (5.9)	5111 (52.9)	1.28
Foley catheter	31,762 (31.3)	8101 (83.9)	1.27
Levin tube	1267 (1.2)	1185 (12.3)	0.50
Patient-controlled analgesia (intravenous/other)	43,863 (43.2)/142 (0.1)	6951 (72.0)/4 (0.0)	0.60
Intraoperative packed red blood cells	0 (0, 0)	0 (0, 1)	0.56
Intraoperative FFP	0 (0, 0)	0 (0, 0)	0.44
Intraoperative PC	0 (0, 0)	0 (0, 0)	0.18
Intraoperative cryoprecipitate	0 (0, 0)	0 (0, 0)	0.05
Rocuronium	40 (30, 50)	50 (40, 90)	0.54
Vecuronium	0 (0, 0)	0 (0, 0)	0.33
Atracurium	0 (0, 0)	0 (0, 0)	0.24
Cisatracurium	0 (0, 0)	0 (0, 0)	0.21
Succinylcholine	0 (0, 0)	0 (0, 0)	0.06
Pyridostigmine	0 (0, 15)	0 (0, 15)	0.12
Neostigmine	0 (0, 2)	0 (0, 2)	0.28
Sugammadex	0 (0, 0)	0 (0, 0)	0.24
Fentanyl	0.04 (0, 1)	0.8 (0, 1.2)	0.53
Alfentanil	0 (0, 0)	0 (0, 0.25)	0.14
Sufentanil	0 (0, 0)	0 (0, 0)	0.30
Remifentanil	1 (0, 1)	1 (1, 2)	0.48
Pethidine	0 (0, 0)	0 (0, 0)	0.02
BUN	13.8 (11.1, 17)	15.4 (12, 20.2)	0.38
Cr	0.8 (0.66, 0.92)	0.81 (0.69, 1)	0.24
GFR	91.3 (78.2, 106.7)	82.8 (65.3, 101.25)	0.27
PT	12.5 (11.8, 13.1)	12.6 (11.7, 13.5)	0.16
aPTT	33.9 (31.3, 36.9)	33.3 (30.1, 37)	0.03
INR	0.99 (0.94, 1.04)	1.02 (0.96, 1.1)	0.29
Platelet count	245 (207, 289)	224 (178, 279)	0.21
Na	140 (138, 141)	139 (137, 141)	0.24
K	4.2 (3.9, 4.4)	4 (3.8, 4.3)	0.28
Uric acid	4.8 (3.9, 5.9)	4.7 (3.6, 5.9)	0.09
Protein	7.2 (6.8, 7.5)	6.8 (6.2, 7.2)	0.62
Albumin	4.4 (4.2, 4.6)	4.1 (3.6, 4.4)	0.77
Robotic surgery	2153 (2.1)	450 (4.7)	0.14
Laparoscopic surgery	17811 (17.5)	1931 (20.0)	0.06
Heart surgery	81 (0.1)	334 (3.5)	0.32
Abdominal surgery (minor/major)	14,959 (14.7)/2362 (2.3)	1274 (13.2)/1441 (14.9)	0.40
Breast surgery (minor/major)	4577 (4.5)/10 (0.0)	31 (0.3)/6 (0.1)	0.29
Ear surgery	2479 (2.4)	22 (0.2)	0.22
Endocrine surgery (minor/major)	1651 (1.6)/1396 (1.4)	68 (0.7)/25 (0.3)	0.16
Eye surgery	2049 (2.0)	16 (0.2)	0.20
Head and neck surgery (minor/major)	12,361 (12.2)/76 (0.1)	132 (1.4)/26 (0.3)	0.43
Musculoskeletal surgery (minor/major)	28,873 (28.4)/1227 (1.2)	1524 (15.8)/665 (6.9)	0.02
Neurosurgery (minor/major)	2787 (2.7)/985 (1.0)	646 (6.7)/55 (0.6)	0.39
OBGY surgery (minor/major)	9486 (9.3)/238 (0.2)	169 (1.8)/55 (0.6)	0.27
Spine surgery (minor/major)	2928 (2.9)/1719 (1.7)	361 (3.7)/480 (5.0)	0.19
Thoracic surgery (minor/major)	262 (0.3)/114 (0.1)	355 (3.7)/281 (2.9)	0.39
Transplantation surgery (minor/major)	25 (0.0)/45 (0.0)	12 (0.1)/50 (0.5)	0.11
Urogenital surgery (minor/major)	8789 (8.7)/575 (0.6)	267 (2.8)/456 (4.7)	0.06
Vascular surgery (minor/major)	630 (0.6)/20 (0.0)	159 (1.6)/61 (0.6)	0.16
Skin and soft tissue surgery (minor/major)	4041 (4.0)/88 (0.1)	114 (1.2)/31 (0.3)	0.13

aPTT = activated partial thromboplastin time, ASD = absolute standardized difference, BUN = blood urea nitrogen, Cr = creatinine, FFP = fresh frozen plasma, GFR = glomerular filtration rate, INR = international normalized ratio, OBGY = obstetric and gynecologic surgery, PC = platelet concentrate, PPCs = postoperative pulmonary complications, PT = prothrombin time.

**Table 3:** Performance metrics of each model for predicting postoperative pulmonary complications.

	Accuracy (95% CI)	Recall (95% CI)	Precision (95% CI)	AUROC (95% CI)	F1 Score (95% CI)
Logistic regression	0.866 (0.861–0.87)	0.647 (0.626–0.669)	0.352 (0.336–0.368)	0.767 (0.756–0.778)	0.456 (0.439–0.472)
Random forest	0.912 (0.908–0.915)	0.564 (0.542–0.587)	0.492 (0.471–0.513)	0.754 (0.743–0.766)	0.526 (0.507–0.544)
MLP neural network	0.899 (0.895–0.903)	0.471 (0.448–0.493)	0.425 (0.404–0.446)	0.705 (0.694–0.716)	0.446 (0.427–0.465)
XG boost	0.924 (0.92–0.927)	0.427 (0.405–0.449)	0.582 (0.556–0.607)	0.699 (0.688–0.71)	0.492 (0.471–0.512)
Light GBM	0.924 (0.921–0.928)	0.452 (0.43–0.475)	0.583 (0.557–0.607)	0.711 (0.699–0.722)	0.509 (0.488–0.53)

AUROC = area under the receiver-operating characteristic curve, CI = confidence interval, GBM = gradient boosting machine, MLP = multilayer perceptron, XG = extreme gradient.

**Table 4:** Performance metrics of models including top 10 features for predicting postoperative pulmonary complications.

	Accuracy (95% CI)	Recall (95% CI)	Precision (95% CI)	AUROC (95% CI)	F1 Score (95% CI)
Logistic regression	0.769 (0.764–0.775)	0.69 (0.67–0.711)	0.227 (0.216–0.238)	0.733 (0.723–0.744)	0.342 (0.328–0.355)
Random forest	0.894 (0.89–0.898)	0.574 (0.553–0.596)	0.419 (0.4–0.438)	0.749 (0.738–0.761)	0.485 (0.466–0.502)
XG boost	0.907 (0.904–0.911)	0.401 (0.379–0.424)	0.461 (0.437–0.485)	0.678 (0.667–0.689)	0.429 (0.408–0.449)
Light GBM	0.916 (0.913–0.92)	0.277 (0.257–0.297)	0.537 (0.506–0.567)	0.627 (0.617–0.637)	0.365 (0.343–0.387)

GBM = gradient boosting machine, XG = extreme gradient, CI = confidence interval, AUROC = area under the receiver-operating characteristic curve.

**Figure A1:**
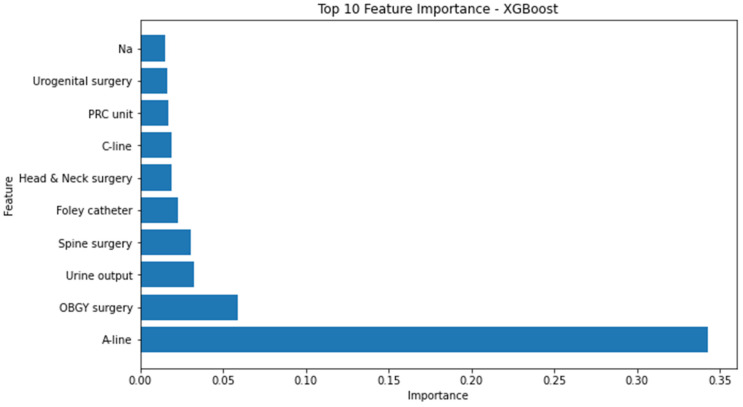
Top 10 feature importance of XG boosting model. A-line = arterial continuous monitoring line, ASA-PS = American Society of Anesthesiologists physical status, C-line = central venous pressure monitoring line, OBGY = obstetrics and gynecology, PRC = packed red cell, XG = extreme gradient.

**Figure A2:**
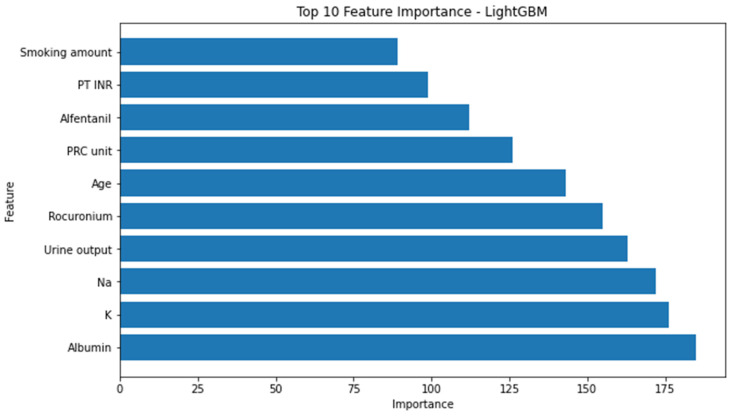
Top 10 feature importance of light-gradient boosting machine model. INR = international normalized ratio, GBM = gradient boosting machine, PT = prothrombin time, PRC = packed red cell.

## Data Availability

Restrictions apply to data availability. Data were obtained from the Hallym Medical Center and are available from its clinical data warehouse, with permission from the Hallym Medical Center.

## Supplementary Materials

The following supporting information can be downloaded at: https://www.mdpi.com/article/10.3390/jcm12175681/s1, Table S1: Methods used to determine pulmonary complications. Table S2: Definitions of demographic characteristics and perioperative covariates.
