# Supplementary material for: Harnessing Machine Learning for Prediction of Postoperative Pulmonary Complications: Retrospective Cohort Design
Source: J Clin Med. 2023 Aug 31;12(17):5681. doi: 10.3390/jcm12175681 (PMC10488713; doi:10.3390/jcm12175681)
Supplement: Supplementary file 1 [file jcm-12-05681-s001.zip › jcm-2524014-supplementary.pdf]

Supplementary Table S1. Methods used to determine pulmonary complications.

Complication	Method
Atelectasis	Chest X-ray.
Pulmonary edema	Chest X-ray.
Pleural effusion	Chest X-ray.
Pneumothorax	Chest X-ray.
Pulmonary embolism	Chest computed tomography.
Respiratory failure	Ventilator dependence for ≥2 postoperative days.
Pneumonia	Pneumonia or infiltration with fever (>38 °C), white blood cell count < 4000/ µL or > 12000/µL or positive blood culture.
Acute respiratory distress	Diagnosis based on respiratory medical consultation.

Supplementary Table S2. Definitions of demographic characteristics and perioperative covariates.

**Patient's characteristics**	
Age	years
Male	Male in medical records.
Order of surgery	Order of surgery received during hospitalization.
Cooperative surgery	1: no Cooperative surgery.<br>>2: number of Cooperative surgery.
Body mass index	Weight (kg)/height (m)^2
Congestive heart failure	KCD code: I09.9, I11.0, I13.0, I13.2, I25.5, I42.0, I42.5- I42.9, I43.x, I50.x, P29.0
Cardiac arrhythmias	KCD code: I44.1-I44.3, I45.6, I45.9, I47.X-I49.X, R00.0, R00.1, R00.8, T82.1, Z45.0, Z95.0
Valvular disease	KCD code: A52.0, I05.X-I08.X, I09.1, I09.8, I34.x-I39.x, Q23.0, Q23.3, Z95.2-Z95.4
Pulmonary circulation disorders	KCD code: I26.x, I27.x, I28.0, I28.8, I28.9
Peripheral vascular disorders	KCD code: I70.x, I71.x, I73.1, I73.8, I73.9, I77.1, I79.0, I79.2, K55.1, K55.8, K55.9, Z95.8, Z95.9
Hypertension, uncomplicated	KCD code: 110.x
Hypertension, complicated	KCD code: I11.x-I13.x, I15.x
Paralysis	KCD code: G04.1, G 11.4, G80.1, G80.2, G81.x, G82.x, G83.0-G83.4, G83.9
Other neurological disorders	KCD code: G10.x-G13.x, G20.x-G22.x, G25.4, G25.5, G31.2, G31.8, G31.9, G32.x, G35.x-G37.x, G40.x, G41.X, G93.1, G93.4, R47.0, R56.x
Chronic pulmonary disease	KCD code: 127.8, 127.9, J40.X-J47.X, J60.X-J67.X, J68.4, J70.1, J70.3
Diabetes, uncomplicated	KCD code: E10.0, E10.1, E10.9, E11.0, E11.1, E11.9, E12.0, E12.1, E12.9, E13.0, E13.1, E13.9, EI4.0, E14.1, E14.9
Diabetes, complicated	KCD code: E10.2-E10.8, E11.2-E11.8, E12.2- E12.8. E13.2-E13.8, E14.2-E14.8
Hypothyroidism	KCD code: E00.X-E03.X, E89.0
Renal failure	KCD code: 112.0, 113.1, N18.x, N19.x, N25.0. Z49.0, Z49.2, Z94.0, Z99.2
Liver disease	KCD code: B18.x, I85.x, I86.4, I98.2, K70.x, K71.1, K71.3-K71.5, K71.7, K72.x- K74.x, K76.0, K76.2 K76.9, Z94.4
Peptic ulcer disease excluding bleeding	KCD code: K.25.7, K.25.9, K26.7, K.26.9, K27.7, K.27.9, K28.7, K28.9
AIDS/HIV	KCD code: B20, B21, B22, B24
Lymphoma	KCD code: C81, C82, C83, C84, C85, C88, C96, C900, C902
Metastatic cancer	KCD code: C77, C78, C79, C80
Solid tumor without metastasis	KCD code: C00, C01, C02, C03, C04, C05, C06, C07, C08, C09, C10, C11, C12, C13, C14, C15, C16, C17, C18, C19, C20,

	C21, C22, C23, C24, C25, C26, C30, C31, C32, C33, C34, C37, C38, C39, C40, C41, C43, C45, C46, C47, C48, C49, C50, C51, C52, C53, C54, C55, C56, C57, C58, C60, C61, C62, C63, C64, C65, C66, C67, C68, C69, C70, C71, C72, C73, C74, C75, C76, C97
Rheumatoid arthritis/ collagen vascular diseases	KCD code: L940, L941, L943, M05, M06, M08, M120, M123, M30, M310, M311, M312, M313, M32, M33, M34, M35, M45, M461, M468, M469
Coagulopathy	KCD code: D65-D68.x, D69.1, D69.3- D69.6
Obesity	KCD code: E66
Weight loss	KCD code: E40, E41, E42, E43, E44, E45, E46, R634, R64
Fluid and electrolyte disorders	KCD code: E22.2, E86.x, E87.x
Blood loss anemia	KCD code: D500
Deficiency anaemia	KCD code: D50.8, D50.9, D51.x-D53.x
Alcohol abuse	KCD code: F10, E52, G62.1, I42.6, K29.2, K70.0, K70.3, K70.9, T51.x, Z50.2, Z71.4, Z72.1
Drug abuse	KCD code: F11.x-F16.x, F18.x, F19.x, Z71.5, Z72.2
Psychoses	KCD code: F20.x, F22.x-F25.x, F28.x, F29.x, F30.2, F31.2, F31.5
Depression	KCD code: F20.4, F31.3-F31.5, F32.x, F33.x, F34.1, F41.2, F43.2
Unconsciousness	Preoperative consciousness evaluation on medical records.
Alcohol	On the electronic medical record, the patient's drinking status item.
Smoking amount	Smoking amount (packs) / day on the electronic medical record.
Smoking duration	Dmoking duration (years) On the electronic medical record
Emergency	Recorded as emergency surgery in pre-anesthesia records.
ASA-PS	Recorded as American Society of Anesthesiologists Physical Status Classification in pre-anesthesia records.
General anesthesia	General anesthesia on anesthesia record
N_2O	Use of N_2O on prescription data for health insurance claims.
Inhalation anesthetics	Use of inhalation anesthetics on prescription data for health insurance claims.
Anesthesia time	Time from start to end of anesthesia.
Surgery time	Time from start to end of surgery.
Intraoperative fluid administration	Administered amount (ml) of total fluid regardless of kinds of fluid during surgery.
Intraoperative urine output	Urine output (ml) during surgery.
Arterial line	Use of arterial line monitoring on prescription data for health insurance claims.

Central venous line	Use of central venous line monitoring on prescription data for health insurance claims.
Foley catheter	Use of foley catheter on prescription data for health insurance claims.
Levin tube	Use of Levin tube on prescription data for health insurance claims.
Patient-controlled analgesia (intravenous/other)	Use of patient-controlled analgesia on prescription data for health insurance claims.
Intraoperative packed red blood cells	Administered amount (unit) of packed red blood cells.
Intraoperative FFP	Administered amount (unit) of FFP.
Intraoperative PC	Administered amount (unit) of PC.
Intraoperative cryoprecipitate	Administered amount (unit) of cryoprecipitate.
Rocuronium	Administered dose of rocuronium during surgery.
Vecuronium	Administered dose of Vecuronium during surgery.
Atracurium	Administered dose of Atracurium during surgery.
Cisatracurium	Administered dose of Cisatracurium during surgery.
Succinylcholine	Administered dose of Succinylcholine during surgery.
Pyridostigmine	Administered dose of Pyridostigmine during surgery.
Neostigmine	Administered dose of Neostigmine during surgery.
Sugammadex	Administered dose of Sugammadex during surgery.
Fentanyl	Administered dose of Fentanyl during surgery.
Alfentanil	Administered dose of Alfentanil during surgery.
Sufentanil	Administered dose of Sufentanil during surgery.
Remifentanil	Administered dose of Remifentanil during surgery.
Pethidine	Administered dose of Pethidine during surgery.
Arterial line monitoring	Use of arterial line monitoring on prescription data for health insurance claims.
Central venous line monitoring	Use of central venous line monitoring on prescription data for health insurance claims.
Foley catheter	Use of foley catheter on prescription data for health insurance claims.
Patient controlled analgesia	Use of patient-controlled analgesia on prescription data for health insurance claims.
BUN (mg/dl)	Most recent preoperative test result for blood urea nitrogen.
Creatinine (mg/dl)	Most recent preoperative test result for creatine.
GFR (ml/min/1.73)	Most recent preoperative test result for glomerular filtration rate.
aPTT (seconds)	Most recent preoperative test result for activated partial thromboplastin time.
PT	Most recent preoperative test result for prothrombin time.
INR	Most recent preoperative test result for international normalized ratio of prothrombin time.

Platelet ($\mu\ell^{-1}$)	Most recent preoperative test result for platelet.
Albumin (g/dl)	Most recent preoperative test result for albumin.
Robotic surgery	Robot-assisted surgery.
Laparoscopic surgery	type of surgical procedure that allows a surgeon to access the inside of the abdomen and pelvis without having to make large incisions in the skin.
Heart surgery	Procedure classification code: O0260, O0881, O1640-49, O1660, O1710-11, O1721-22, O1730, O1781-82, O1792-99, O1821, O1823, O1840-41, O1890, O1901-07, O1910, O1921-22, O1931-32, O1940, O1950, O1970, O1981-2, O2004, O2006, O2211, OA640-1, OA647-9
Abdomen surgery	Procedure classification code: Q025x, Q0292, Q126x, Q2533-Q2534, Q2537, Q259x, Q2630-Q2638, Q2671-Q2673, Q2679, Q2687-Q2688, Q2921-Q2926, Q2928, Q2982-Q2984, Q722x-Q723x, Q7342, Q7360-Q7371, Q7410, Q7561-Q7562, Q7564, Q7567, Q757x, QA67x, QA92x, P208x-P209x, Q244x-Q252x, Q2536, Q254x-Q257, Q260x-Q262x, Q2639, Q264x-Q266x, Q2676, Q2680, Q269x-Q291x, Q2927, Q293x-Q297x, Q2981, Q299x-Q306x, Q721x, Q724x-Q733x, Q7341, Q7351-Q7352, Q7372, Q738x-Q740x, Q742x-Q755x, Q7563, Q7565-Q7566, Q758x-Q779x, QA536, QA63x-QA64x, QA75x, QX706, QX891
Musculoskeletal surgery	Procedure classification code: N0408, N0571, N0587, N0704, N0708, N0715, N1583-N1584, N1711, N1715, N1721, N1725, N2070, N2710-N2711, N2716, N3710-N3712, N3716-N3717, N3720-N3722, N3726-N3727, N4710-N4712, N4716-N4717, N4720-N4722, N4726-N4727, N002x, N025x-N031x, N035x-N039x, N0402-N0407, N041x-N043x, N050x-N056x, N0572-N0584, N0588, N059x-N062x, N0631-N0635, N064x-N069x, N0700-N0703, N0705-N0706, N0709-N0710, N0714, N0717-N0719, N072x-N100x, N1581-N1582, N1585, N160x-N161x, N1714, N1717, N1724, N1727, N2071-N2079, N2712-N2715, N2717-N2719, N3713-N3715, N3718-N3719, N3723-N3725, N3728-N3279, N4713-N4715, N4718-N4719, N4723-N4725, N4728-N4729, NA28x, NY05x
Neurosurgery	Procedure classification code: S4621-S4622, S4634-S2639, S464x-S466x, S4671, S4681, S4684, S4694-S4696, S4704, S4706, S4708, S4713, S4721, S4733-S4737, S4760, S4780, S4799, S4801-S4803, S6692, S6694, S6696, N032x-N034x, S047x, S459x-S461x, S4625, S4670, S4682-S4683, S4685, S4705, S4707, S4709-S4712, S4722-S4732, S474x-S475x, S477x, S4792-S4798, S4805, S481x-S485x, S6691, S6693, S6695, SY62x-SY63x
OBGY surgery	Procedure classification code: R014x, R040x-R041x, R313x-R314x, R401x-R500x, RA31x, RA36x-RA38x, RA43x, RY54x, RZ56x
Spine surgery	Procedure classification code: N0455, N0466, N0471-N0480, N1460, N1469, N2464-N2470, N044x, N0451-N0454, N0468-

	N0469, N0630, N1466, N149x, N2461-N2463, N2471-N2472, N249x
Thoracic surgery	Procedure classification code: O130x, O1313-O1319, O132x-O162x O160x-O162x, Q233x-Q243x, QA42x
Vascular surgery	Procedure classification code: O016x-O017x, O0215-O2018, O0223-O0227, O026x-O028x, O1635, O1643-O1646, O165x, O201x-O208x, O265x, OA63x, OA65x, OB63x-OB64x, OX181, OZ201
Skin & soft tissue surgery	Procedure classification code: SA161-SA165, SC161-SC165, N001x, N004x-N024x, N113x, N151x, NA05x, NA24x, NX201, S016x-S017x, SB16x-SB17x, SB27x
Major operation surgery	Procedure classification code: Q025x, Q0292, Q126x, Q2533-Q2534, Q2537, Q259x, Q2630-Q2638, Q2671-Q2673, Q2679, Q2687-Q2688, Q2921-Q2926, Q2928, Q2982-Q2984, Q722x-Q723x, Q7342, Q7360-Q7371, Q7410, Q7561-Q7562, Q7564, Q7567, Q757x, QA67x, QA92x, N0408, N0571, N0587, N0704, N0708, N0715, N1583-N1584, N1711, N1715, N1721, N1725, N2070, N2710-N2711, N2716, N3710-N3712, N3716-N3717, N3720-N3722, N3726-N3727, N4710-N4712, N4716-N4717, N4720-N4722, N4726-N4727, S4621-S4622, S4634-S2639, S464x-S466x, S4671, S4681, S4684, S4694-S4696, S4704, S4706, S4708, S4713, S4721, S4733-S4737, S4760, S4780, S4799, S4801-S4803, S6692, S6694, S6696, N0455, N0466, N0471-N0480, N1460, N1469, N2464-N2470, SA161-SA165, SC161-SC165

KCD = Korean Standard Classification of Diseases (https://www.kcdcode.kr/browse/main/, accessed on 19 September 2022)

Procedure classification code (https://www.koicd.kr/ins/act.do, 19 September 2022)

aPTT = activated partial thromboplastin time, ASA-PS = American Society of Anesthesiologists physical status, BUN = blood urea nitrogen, INR = international normalized ratio, OBGY = obstetrics and gynecology, AIDS = acquired immune deficiency syndrome FFP = fresh frozen plasma, GFR = glomerular filtration rate, HIV = human immunodeficiency virus, PC = platelet concentrate, PT = prothrombin time.
